# Supplementary material for: Modulation of neural oscillations during working memory update, maintenance, and readout: An hdEEG study
Source: Hum Brain Mapp. 2020 Nov 17;42(4):1153–66. doi: 10.1002/hbm.25283 (PMC7856639; doi:10.1002/hbm.25283)
Supplement: Supplementary file 1 — Figure S1 Effect of PHASE in the γLOW and γHIGH band. Violin plots of ERS/ERD variation in the γLOW (yellow) and γHIGH (orange) bands during update (top), maintenance (middle) and readout (bottom). Superimposed in gray are boxplots describing the median value (white dot), 25th and 75th percentiles (extremes of the thick gray line), and full data range (extremes of the thin gray line) of the distributions. Table S1: results of ANOVA main effect of TRIAL. Asterisks report the level of significance (** p < 0.01; * p < 0.05). Table S2. Results of ANOVA related to the interaction of TASK*PHASE in the γLOW band. Asterisks report the level of significance (** p < 0.01; * p < 0.05; $ trend). U, update; M, maintainance; R, readout. In the descriptive statistics, mean ± standard deviation is reported. Table S3. Results of ANOVA related to the interaction of interaction of TRIAL*PHASE in the γLOW band. Asterisks report the level of significance (** p < 0.01; * p < 0.05; $ trend). U, update; M, maintainance; R, readout. TP, true positive trials; TN, true negative trials. In the descriptive statistics, mean ± standard deviation is reported. Table S4. Results of ANOVA related to interaction of TRIAL*PHASE*TASK in the γLOW band. Asterisks report the level of significance (** p < 0.01; * p < 0.05; $ trend). U, update; M, maintainance; R, readout. TP, true positive trials; TN, true negative trials. In the descriptive statistics, mean ± standard deviation is reported. [file HBM-42-1153-s001.docx]

### Effect of TRIAL

Table 1S shows the results of ANOVA for TRIAL as main effect. We found that TP and TN trials were respectively characterized by an increase and decrease of oscillations. This effect was found significant (p ≤ 0.04) in the β band for right hemisphere and both cerebellar ROIs, while in γ bands for left subcortical and cerebellar ROIs. We also found a significant effect (p = 0.035) for PPC-R in the γ_LOW_ band only.

**Figure 1S**

**
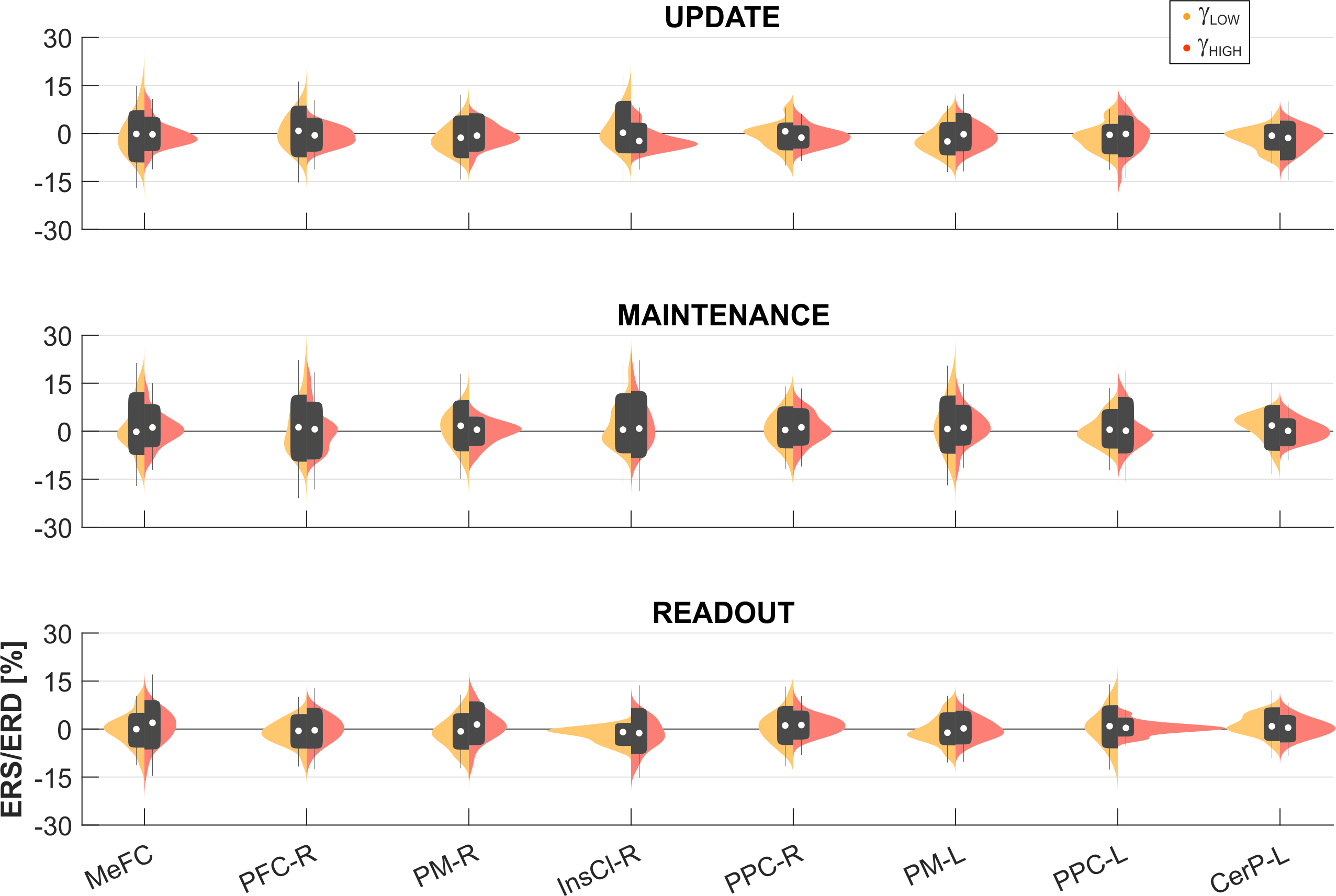
**

**Figure 1S:** Effect of PHASE in the γ_LOW_ and γ_HIGH_ band. Violin plots of ERS/ERD variation in the γ_LOW_ (yellow) and γ_HIGH_ (orange) bands during update (top), maintenance (middle) and readout (bottom). Superimposed in grey are boxplots describing the median value (white dot), 25th and 75th percentiles (extremes of the thick grey line), and full data range (extremes of the thin grey line) of the distributions.

**Table 1S:** results of ANOVA main effect of TRIAL. Asterisks report the level of significance (** p<0.01; * p<0.05).

| ROI | θ | β | γ_LOW_ | γ_HIGH_ |
| --- | --- | --- | --- | --- |
| MeFC | F_1.20_ = 0.038  p = 0.84 | F_1.20_ = 4.36*  p = 0.04 | F_1.20_ = 1.06  p = 0.31 | F_1.20_ = 0.009  p = 0.92 |
| PFC-R | F_1.20_ = 1.92  p = 0.18 | F_1.20_ = 14.24**  p = 0.001 | F_1.20_ = 1.47  p = 0.23 | F_1.20_ = 1.23  p = 0.28 |
| PMC-R | F_1.20_ = 1.92  p = 0.18 | F_1.20_ = 14.24**  p = 0.001 | F_1.20_ = 1.47  p = 0.23 | F_1.20_ = 1.23  p = 0.28 |
| InsCl-R | F_1.20_ = 1.00  p = 0.32 | F_1.20_ = 13.18**  p = 0.001 | F_1.20_ = 2.15  p = 0.15 | F_1.20_ = 0.35  p = 0.55 |
| PPC-R | F_1.20_ = 0.09  p = 0.76 | F_1.20_ = 2.47  p = 0.13 | F_1.20_ = 5.08  p = 0.035 | F_1.20_ = 3.26  p = 0.08 |
| CerT-R | F_1.20_ = 0.008  p = 0.92 | F_1.20_ = 7.63*  p = 0.01 | F_1.20_ = 1.81  p = 0.19 | F_1.20_ = 2.14  p = 0.15 |
| DLPFC-L | F_1.20_ = 0.27  p = 0.61 | F_1.20_ = 0.05  p = 0.81 | F_1.20_ = 0.009  p = 0.92 | F_1.20_ = 1.51  p = 0.23 |
| FC-L | F_1.20_ = 0.06  p = 0.79 | F_1.20_ = 0.06  p = 0.80 | F_1.20_ = 0.003  p = 0.95 | F_1.20_ = 0.04  p = 0.83 |
| PMC-L | F_1.20_ = 1.79  p = 0.19 | F_1.20_ = 0.28  p = 0.60 | F_1.20_ = 0.17  p = 0.68 | F_1.20_ = 0.0009  p = 0.97 |
| InsCl-L | F_1.20_ = 0.15  p = 0.70 | F_1.20_ = 3.84  p = 0.06 | F_1.20_ = 0.03  p = 0.86 | F_1.20_ = 2.47  p = 0.13 |
| PPC-L | F_1.20_ = 0.009  p = 0.93 | F_1.20_ = 2.60  p = 0.12 | F_1.20_ = 1.48  p = 0.23 | F_1.20_ = 3.25  p = 0.08 |
| Fus-L | F_1.20_ = 1.67  p = 0.21 | F_1.20_ = 1.35  p = 0.25 | F_1.20_ = 0.96  p = 0.33 | F_1.20_ = 5.91*  p = 0.024 |
| CerT-L | F_1.20_ = 0.35  p = 0.55 | F_1.20_ = 3.01  p = 0.097 | F_1.20_ = 7.56*  p = 0.012 | F_1.20_ = 6.93*  p = 0.015 |
| CerP-L | F_1.20_ = 0.033  p = 0.85 | F_1.20_ = 5.21*  p = 0.033 | F_1.20_ = 4.94*  p = 0.037 | F_1.20_ = 3.15  p = 0.09 |

### Interactions of main effects

**Table 2S.** Results of ANOVA related to the interaction of TASK*PHASE in the γ_LOW_ band. Asterisks report the level of significance (** p<0.01; * p<0.05; ^$^ trend). U, update; M, maintainance; R, readout. In the descriptive statistics, mean ± standard deviation is reported.

| ROI | Within test | Descriptive statistics | Post-hoc |
| --- | --- | --- | --- |
| CerT_R | F_2.40_ = 7.13**  p = 0.002 | U: 2-back (-0.97 ± 1.13) 3-back (-3.88 ± 0.95)  M.: 2-back (-0.84 ± 1.16) 3-back (-0.86 ± 1.12)  R: 2-back (-2.35 ± 0.95) 3-back (-0.39 ± 1.39) | p = 0.01*  p = 0.25  p = 0.16 |
| CerP_L | F_2.40_ = 3.58*  p = 0.036 | U: 2-back (-0.32 ± 1.20) 3-back (-2.84 ± 0.82)  M.: 2-back (0.79 ± 1.75) 3-back (0.05 ± 1.20)  R: 2-back (-0.80 ± 0.91) 3-back (1.12 ± 1.28) | p = 0.09^$^  p = 0.09^$^  p = 0.7 |

**Table 3S.** Results of ANOVA related to the interaction of interaction of TRIAL*PHASE in the γ_LOW_ band. Asterisks report the level of significance (** p<0.01; * p<0.05; $ trend). U, update; M, maintainance; R, readout. TP, true positive trials; TN, true negative trials. In the descriptive statistics, mean ± standard deviation is reported.

| ROI | Within test | Descriptive statistics | Post-hoc |
| --- | --- | --- | --- |
| CerT_L | F_2.40_ = 4.76*  p = 0.013 | U: TP (-3.42 ± 1.26) TN (-1.88 ± 0.88)  M: TP (0.84 ± 1.86) TN (-0.87 ± 0.55)  R: TP (2.91 ± 1.24) TN (-2.44 ± 0.82) | p = 0.2  p = 0.3  p = 0.0005** |
| Fus_L | F_2.40_ = 6.96**  p = 0.002 | U: TP (-4.40 ± 1.42) TN (-1.15 ± 0.80)  M: TP (0.41 ± 1.83) TN (-0.53 ± 0.64)  R: TP (3.13 ± 1.42) TN (-1.98 ± 0.75) | p = 0.03*  p = 0.9  p = 0.0012** |

**Table 4S**. Results of ANOVA related to interaction of TRIAL*PHASE*TASK in the γ_LOW_ band. Asterisks report the level of significance (** p<0.01; * p<0.05; $ trend). U, update; M, maintainance; R, readout. TP, true positive trials; TN, true negative trials. In the descriptive statistics, mean ± standard deviation is reported.

| ROI | Within test | Descriptive statistics | Post-hoc |
| --- | --- | --- | --- |
| CerT-R | F_2.40_ = 4.47*  p = 0.017 | TP:  U: 2-back(0.71 ± 2.07) 3-back (-5.36 ± 1.91)  M: 2 back (0.98 ± 2.27) 3-back (-0.27 ± 1.73)  R.: 2-back(-1.74 ± 1.54) 3-back (2.09 ± 2.01)  TN:  U: 2-back(-2.65 ± 0.8) 3-back (-2.39 ± 1.02)  M.: 2 back (0.69 ± 0.94) 3-back (1.4 ± 1.03)  R: 2-back (-2.94 ± 0.78) 3-back (-2.87 ± 1.03) | p = 0.006**  p = 0.64  p = 0.048*  p = 0.80  p = 0.11  p = 0.95 |
| Fus-L | F_2.40_ = 3.70*  p = 0.03 | TP:  U: 2-back (-0.29 ± 1.81) 3-back (-8.5 ± 2.32)  M: 2-back (-0.96 ± 2.61) 3-back (0.14 ± 2.05)  R: 2-back (3.28 ± 1.62) 3-back (2.98 ± 1.93)  TN:  U: 2-back (-0.84 ± 1.08) 3-back (-1.44 ± 1.00)  M: 2-back (0.26 ± 0.85) 3-back (-1.32 ± 0.78)  R: 2-back (-1.29 ± 0.92) 3-back (-2.66 ± 0.95) | p = 0.014*  p = 0.71  p = 0.89  p = 0.66  p = 0.13  p = 0.24 |
